# Establishing an implementation framework for clinical nursing guidelines in hepatobiliary and accelerated rehabilitation surgery: based on the Ottawa research application model

**DOI:** 10.3389/fsurg.2026.1714963

**Published:** 2026-02-23

**Authors:** Ting Dai, Honghui Zhang, Yuting Xiao

**Affiliations:** 1 Department of Nursing, Hunan Provincial People's Hospital, The First-Affiliated Hospital of Hunan Normal University, Hunan, China; 2 Department of Hepatobiliary Surgery, Hunan Provincial People's Hospital, The First-Affiliated Hospital of Hunan Normal University, Hunan, China

**Keywords:** clinical nursing practice guideline, hepatological surgery department, implementation of guideline, Ottawa model of research use, Ottawa research application model

## Abstract

**Objective:**

To establish a clinical nursing practice guideline for Accelerated Rehabilitation in Hepatobiliary Surgery (ERAS) based on the Ottawa research application model.

**Methods:**

Using the Ottawa research application model as the guide, the obstacle factors were analyzed through focus group interview, and the comprehensive intervention strategy was developed by expert consultation.

**Results:**

10 barrier factors were identified from three aspects: evidence, adopters and practice environment, and multi-dimensional intervention strategies were constructed, including the Manual of ERAS Nursing Management in Hepatobiliary Surgery, telemedicine platform and multidisciplinary collaboration process.

**Conclusion:**

The Ottawa model provides a systematic framework for the application of guidelines, and leadership support and technology integration are the keys to practice. This study provides reference for standardization of ERAS nursing practice and resource optimization.

## Introduction

Hepatobiliary and cholangiocarcinoma diseases typically include conditions such as cholelithiasis, gallbladder polyps, bile duct stones, and hepatocellular carcinoma. These disorders are characterized by frequent emergency cases, rapid disease progression, high surgical complexity, and severe complications that often lead to fatal outcomes, posing significant threats to patients ‘lives (1). Research indicates that the incidence of most hepatobiliary and cholangiocarcinoma diseases is on the rise (2–4). The expanding scale of these conditions not only endangers patients’ health but also imposes substantial economic burdens on families and society (5, 6).Enhanced Recovery After Surgery (ERAS), first proposed by Danish surgeon Henrik Kehlet, has become a widely adopted clinical practice. Its core philosophy integrates evidence-based measures to optimize perioperative care pathways through multidisciplinary collaboration, thereby reducing psychological and physiological stress responses in patients, minimizing surgical trauma and stress-related damage, and accelerating postoperative rehabilitation (7). The emergence of perioperative ERAS provides scientifically validated nursing interventions, serving as an effective initiative to improve perioperative medical service quality and conserve healthcare resources (8). With continuous advancements in surgical equipment and techniques, the scope and outcomes of hepatobiliary surgery have been progressively enhanced (9, 10).The Chinese Medical Association's Surgical Branch and Anesthesiology Branch have standardized the ERAS management pathways for hepatobiliary surgery and related fields, which were updated in 2021 as the “Chinese Clinical Practice Guidelines for Accelerated Rehabilitation Surgery (2021 Edition)” (11). However, in the nursing field, there is still a lack of evidence-based “Clinical Nursing Practice Guidelines for Accelerated Rehabilitation Surgery in Hepatobiliary Diseases”.Based on this, guided by the Ottawa Research Application Model (12), this study constructed a practical plan with clinical applicability and operability based on the analysis of barriers, and provided intervention strategies for promoting the application of the guidelines in clinical practice.

## 2 Research technique

### 2.1 Guiding methodology

This study adopts the Ottawa Model of Research Use (OMRO) as its theoretical framework (12), structured into three phases: assessment (barrier analysis), monitoring (strategy formulation), and evaluation (implementation effectiveness). Developed by Jo Logan and Ian D. Graham at the University of Ottawa, Canada, this model focuses on advancing the practical application of research findings. The framework divides the research application process into three stages: assessment, monitoring, and evaluation. During the assessment phase, researchers must analyze potential implementation barriers from three perspectives: evidence, potential adopters, and practical environments. The monitoring phase involves formulating intervention strategies based on identified barriers to ensure effective evidence application. Guided by this model, the study identifies barrier factors and develops concrete implementation plans to promote the application of the guidelines.

### 2.2 Specific methods

#### 2.2.1 Methodology for barrier analysis

This study employed focus group interviews through purposive sampling, selecting 35 professionals involved in hepatobiliary surgical disease management and nursing care from three hospitals. Eligibility Criteria: (1) Must be employed in hepatobiliary surgery departments (including clinical nursing, surgical care, clinical nutrition, or department management) at Grade III Class A hospitals with ≥5 years of relevant experience (ensuring sufficient practical experience to address guideline implementation challenges); (2) Directly involved in accelerated rehabilitation surgery (ERAS) clinical practices (e.g., postoperative care, patient education, multidisciplinary meetings) or responsible for ERAS quality control in the department; (3) Familiar with the current status of ERAS guideline implementation at the hospital and able to clearly articulate specific challenges encountered in practice; (4) Willing to participate in interviews, demonstrating strong communication skills to ensure complete and authentic feedback.Exclusion criteria: (1) Individuals exclusively engaged in administrative support, research assistance, or other non-ERAS clinical/management roles; (2) Those who have not participated in ERAS practices in hepatobiliary surgery within the past year and lack adequate understanding of guideline implementation; (3) Participants unable to complete the full interview (60–90 min) due to scheduling conflicts or language barriers.Each hospital contributed 11–12 experts. Using semi-structured interview outlines, three focus group sessions were conducted, with content analysis applied to collected data. This study employed Colaizzi's seven-step qualitative analysis method for interview data, which emphasizes “systematically extracting themes from raw materials to ensure results align with participants’ authentic experiences.” The specific steps and quality control measures are as follows:

-Step 1: Familiarize yourself with the materials

Within 24 h after the interview, two researchers trained in qualitative research independently transcribed the audio recordings into transcripts. After transcription, they thoroughly reviewed the materials through a process of “repeated reading, annotating key sentences, and recording initial reflections”.

-Step 2: Identify meaningful units

Extract sentences or paragraphs directly related to “barriers to implementing the ERAS guidelines” from the original text and define them as “meaning units”.

-Step 3: Encoding

The two researchers coded the meaning units independently and assigned the same code to similar content.

-Step 4: Cluster topics

Based on the three-dimensional framework of “Evidence-Acceptor-Practice Environment” of the Ottawa Model of Research Application (OMRO), the initial coding was classified and merged to form 24 sub-topics.

-Step 5: Provide a detailed description

For each subtopic, provide a concrete description using typical statements from the original text to ensure the topic is supported by data.

-Step 6: Verify the subject

Member checking was used to validate the accuracy of the themes: 6 interviewees were invited to read the refined themes and corresponding original statements to confirm their alignment with their actual experiences; the wording of 2 sub-themes was adjusted based on their feedback.

-Step 7: Extract the core theme

The verified sub-topics were further refined to identify the ‘core contradictions hindering guideline implementation,’ ultimately distilling 10 key obstacle themes.Through this process, the study identified three key barriers encountered during implementation of the “Clinical Nursing Practice Guidelines for Accelerated Recovery in Hepatobiliary Surgery Perioperative Care": evidence availability, potential adopters, and practical implementation environments.

#### 2.2.2 Development of application protocols for guidelines

This study employed expert consultation methods to develop application protocols for the guidelines. Specifically, two specialized task forces were established: a guideline application protocol development team and a consultation panel, comprising 12 members in total. All members held bachelor's degrees or higher and possessed relevant experience in hepatobiliary surgical disease treatment, nursing care, or research. The development team consisted of two guideline methodology experts and two research investigators who, based on prior analysis of identified barriers and existing clinical nursing protocols, transformed the guideline recommendations into practical implementation frameworks. These were then refined through feedback from the consultation panel. The consultation panel comprised two hepatobiliary surgical nursing managers, two specialized nurses, two surgical nursing staff, one nutritionist, and one hepatobiliary surgeon. Guided by the FAME principle (13) (Feasibility, Appropriateness, Clinical Significance, Effectiveness), they provided targeted modifications to the protocol drafts proposed by the development team, ultimately finalizing the application protocol.

## 3 Results

### 3.1 Barriers to guideline implementation

The focus group interviews revealed ten major barriers in three key areas when implementing clinical nursing practice guidelines for hepatobiliary surgery: evidence availability, potential adopters, and practical implementation environment (see Table 1). Regarding evidence (recommendations), challenges included complex application of evidence and limited applicability. For potential adopters (practitioners and patients), obstacles comprised inadequate team collaboration among healthcare providers, insufficient patient education, poor compliance rates, and insufficient training for medical staff. Practical implementation barriers encompassed staffing shortages, inadequate equipment resources, entrenched traditional mindsets, lack of innovation culture, complex operational procedures, difficulties in nurse execution, and insufficient multidisciplinary coordination.

**Table 1:** Analysis of barriers to the application of the guidelines and corresponding intervention strategies.

Source	Barriers	Frequency of mentions (*n* = 35)	Percentage (%)	Intervention study
Evidence	Evidence application is highly complex	28	80.0	Based on the guidelines and recommendations, a best practice manual for accelerated rehabilitation nursing management of hepatobiliary surgical diseases was constructed
	The evidence is of limited applicability	25	71.4	Based on the guidelines and recommendations, a best practice manual for accelerated rehabilitation nursing management of hepatobiliary surgical diseases was constructed
	Update and timeliness constraints	23	65.7	Not included in the application of this guide
Adoptor	There is a lack of teamwork among medical staff	21	60.0	Develop a best practice manual for accelerated rehabilitation nursing management of hepatobiliary surgical diseases
	Patients have insufficient education and poor compliance	19	54.3	Develop a self-management manual for accelerated recovery of surgical diseases in hepatobiliary surgery Establish telemedicine, online platforms and VR (virtual reality) technology
	There is insufficient training for health workers	16	45.7	Develop a best practice manual for accelerated rehabilitation nursing management of hepatobiliary surgical diseases
Practical environment	Insufficient manpower and equipment resources	12	34.3	Not included in the application of this guide
	Traditional ideas are deeply rooted and there is no culture of innovation	10	28.6	Not included in the application of this guide
	The process is complex and difficult for nursing staff to implement	8	22.9	Develop a best practice manual for accelerated rehabilitation nursing management of hepatobiliary surgical diseases Train hepatobiliary surgery nurses
	The lack of multi-disciplinary collaboration	12	34.3	Develop a best practice manual for accelerated rehabilitation nursing management of hepatobiliary surgical diseases

### 3.2 Application of the guide

According to the barriers identified in the focus group interviews, the application program construction team and the advisory team of the guide determined the corresponding targeted comprehensive intervention strategies. See Table 1.

#### 3.2.1 Evidence-based considerations for implementation guidelines

The guideline application team conducted rigorous evidence screening, strictly adhering to the principle of evidence availability. During this process, they excluded evidence with high implementation complexity (e.g., guidelines containing multiple management measures), limited applicability (as some guidelines and consensus statements may inadequately address regional or hospital-specific conditions), and evidence constrained by updates and timeliness (e.g., ERAS guidelines and consensus statements requiring continuous revisions that healthcare providers might miss). Through this screening, the team ultimately removed four recommendations from the “Chinese Clinical Practice Guidelines for Accelerated Rehabilitation Surgery (Hepatobiliary Surgery Section)” and incorporated 13 recommendations as evidence sources for this guideline implementation. This study is based on the “China Accelerated Rehabilitation Surgery Clinical Practice Guidelines (2021 Edition · Hepatobiliary Surgery Section)” and combines the results of previous obstacle analysis to screen recommendations according to the three-dimensional principle of “scientific evidence+clinical feasibility + practical applicability”. The specific logic is as follows:

1 The criteria for selecting the 13 recommendations

The 13 recommendations included need to meet all three criteria:

-Standard 1: High-evidence level: All evidence stems from the “Strong Recommendation (1A/1B)” in the guidelines, supported by systematic reviews/Meta-analyses and multicenter randomized controlled trials (RCTs), ensuring scientific rigor and reliability.
-Standard 2: Clinical feasibility: compatible with existing resources (personnel, equipment, processes) at the 3 participating hospitals, without requiring additional special equipment purchases or major adjustments to the existing nursing system.
-Standard 3: Core Coverage: This standard encompasses all ERAS perioperative care phases in hepatobiliary surgery, spanning four key stages: preoperative (patient education and preparation), intraoperative (temperature management), postoperative (pain control, early feeding, rehabilitation, and complication prevention), and post-discharge (follow-up management). It ensures the recommendations form a “nursing closed loop” to address critical clinical needs.

The four rejected recommendations were all deemed to lack “clinical feasibility” or “practical applicability”, with the following specific reasons:

-Excluded recommendation 1: “Use ultrasound-guided transversalis abdominis plane block (TAP) for analgesia within 24 h postoperatively”

Ruling rationale: The evidence's applicability is limited. Although classified as Level 1B evidence, the recommendation is problematic as all three participating hospitals lack dedicated ultrasound analgesia physicians. Moreover, nurses require additional training in TAP block coordination (the current staffing lacks training capacity). Compelling this procedure may cause delayed analgesia or operational risks, thus failing to meet the’ operability’ principle.

-Recommendation 2 excluded: “Perform daily dynamic monitoring of liver and kidney function for all patients after surgery (for 7 consecutive days)”

Reason for exclusion: The implementation is complex and resource-intensive. While this recommendation is necessary for high-risk patients (e.g., cirrhosis or liver resection>50%), it imposes an additional workload of two nurses per day for daily monitoring in mild cases (e.g., gallbladder polyp removal) – a requirement that contradicts the ERAS resource optimization goal. Therefore, it is excluded based on the hierarchical management principle.

-Recommendation 3: “Nutritionists should develop a personalized enteral nutrition plan (including formulation selection) one day before surgery”

Reasons for exclusion: Practical constraints. The hepatobiliary surgery dietitians at the three hospitals operate under a ‘multi-departmental sharing’ model (each managing 3–4 departments). Preoperative individualized treatment plans for all patients require an additional 8–10 h per day, far exceeding current staffing capacity and making routine implementation unfeasible.

Building on this evidence-based approach, the team further adhered to the principle of operational feasibility, closely integrating the selected recommendations with hospital nursing practices to develop the “Best Practice Manual for Accelerated Rehabilitation Nursing Management in Hepatobiliary Diseases”. This manual contains six key sections: (1) Practical Context: Outlines the significance, objectives, and target audience of the manual to provide clear guidance for nursing staff; (2) Evidence Sources: Concisely explains literature search procedures and adopts an evidence hierarchy system to ensure scientific validity and reliability. (3) Evidence Summary: The manual presents the recommended guidelines and their confidence levels from the “Clinical Nursing Practice Guidelines for Accelerated Rehabilitation in Hepatobiliary Surgery” in tabular format, enabling nurses to quickly reference and understand them. (4) Practical Framework & Content: This section details nursing management practices for outpatient and inpatient hepatobiliary surgery departments, covering five key components: target populations, management personnel, operational models, care protocols, and routine practices. It clarifies specific implementation methods, involved personnel, and core elements to guide nurses in effectively applying evidence-based approaches in clinical settings. (5) Operational Tools & Procedures: The manual provides clear, user-friendly workflows and tools for clinical practice, including blood glucose screening protocols, specialized nurse-led management procedures, patient care protocols, and personalized dietary prescriptions for hepatobiliary diseases, ensuring efficient and standardized care. (6) References: The manual lists supporting literature from the evidence source section, offering resources for further study. Through these meticulously designed steps and content arrangements, the handbook aims to provide comprehensive, practical guidance for nurses to better apply evidence in clinical practice, enhance nursing quality, and promote patient recovery.

#### 3.2.2 Adopter-level barriers addressed by practitioners

The guideline application team developed a training program to enhance nurses ‘knowledge and skills in managing hepatobiliary surgical diseases, including group training sessions, case-specific nursing rounds, and clinical discussions. To address patient barriers, the intervention strategies include: (1) Guideline Application Team: Based on the guidelines, they created the “Self-Management Manual for Accelerated Recovery Surgery in Hepatobiliary Diseases,” covering eight key areas: (a) Education and Information Provision (delivering detailed disease information, explaining core concepts of accelerated recovery surgery, helping patients understand its importance, detailing preoperative, intraoperative, and postoperative procedures to reduce anxiety), (b) Preoperative Preparation (psychological and physiological readiness, preoperative evaluations), (c) Postoperative Self-Management (vital signs monitoring, pain management, wound care, dietary guidance), (d) Rehabilitation Tools and Resources (rehabilitation schedules, self-monitoring tools), (e) Family Support and Engagement, (f) Psychological Counseling, (g) Long-term Rehabilitation Plans, and (h) Health Promotion and Lifestyle Changes. These strategies aim to provide comprehensive support for patients to better manage themselves before, during, and after surgery, thereby accelerating recovery. (2) Telemedicine Implementation: Through remote monitoring devices, healthcare providers can track patients’ health status in real-time, identify issues promptly, and adjust treatment plans. Video or text-based remote rehabilitation guidance ensures patients receive professional care support even at home. Additionally, patients can consult doctors remotely via video conferencing software, reducing the inconvenience of hospital visits. (3) Establish an online platform where patients and family members can register accounts simultaneously with role-based access control. The platform provides abundant educational resources including video lectures, illustrated tutorials, and case analyses to help patients and families better understand their conditions and recovery processes. Furthermore, it offers real-time health monitoring tools such as synchronized data from blood pressure monitors and glucose meters, enabling both patients and healthcare providers to track health status in real time. (4) Implement virtual reality (VR) and augmented reality (AR) technologies. VR technology creates immersive rehabilitation training environments, enhancing patient engagement and treatment outcomes. It also provides virtual psychological therapy scenarios to help alleviate anxiety and fear. AR technology further enhances understanding of diseases and rehabilitation processes, such as demonstrating surgical procedures or correct rehabilitation postures through AR visualization.

#### 3.2.3 Practical environment level: addressing barriers in clinical practice

The clinical practice guidance team established specialized nursing teams led by hepatobiliary surgery nurses, with participation from ward responsibility nurses. This initiative aims to address staffing shortages by engaging ward nurses in surgical care management. The “Best Practices Manual for Accelerated Recovery Nursing Management in Hepatobiliary Surgery” was developed to standardize nursing protocols and procedures, ensuring consistent quality of care. Targeted training programs were implemented to equip nurses with new skills and techniques. A multidisciplinary collaboration framework for accelerated recovery was established, clarifying responsibilities across specialties to minimize coordination challenges. The team also proposed optimization strategies for nursing management, actively participating in hospital policy development to promote innovative practices.

## 4 Discussion

### 4.1 The Ottawa research application model provides a framework for the development of application programs for the guide

The Ottawa Research Application Model, as a well-established theoretical framework, provides systematic guidance for developing implementation protocols in this study. This model aims to establish and implement application protocols for the “Clinical Nursing Practice Guidelines for Accelerated Recovery Surgery in Hepatobiliary Diseases”. Hepatobiliary diseases are characterized by frequent emergency cases, rapid disease progression, and high surgical complexity, which significantly impact patient safety. Accelerated Recovery Surgery (ERAS), an emerging perioperative management concept, optimizes perioperative care pathways to reduce physical and psychological stress in surgical patients and accelerate postoperative recovery. However, there remains a lack of evidence-based clinical nursing guidelines for hepatobiliary diseases in the nursing field. Therefore, this study holds significant theoretical and practical importance. In summary, the Ottawa Research Application Model offers a comprehensive framework that enables researchers to systematically identify challenges, design intervention strategies, and evaluate implementation effectiveness. This systematic research methodology not only enhances the scientific rigor and practical value of guideline application but also provides valuable references for future studies and clinical practice.

### 4.2 Systematicness and innovativeness of intervention strategies

Guided by the Ottawa Research Application Model, this study developed a multi-dimensional intervention strategy addressing barriers at three levels: evidence, adopters, and practice environments. At the evidence level, we created the “Best Practice Manual for Accelerated Rehabilitation Nursing Management in Hepatobiliary Surgery” through screening and adaptation of recommended practices. This approach not only simplified complex evidence but also enhanced operational feasibility for nursing staff through clear practical tools and process design. This strategy aligns with Logan et al.'s concept of “knowledge translation tool development,” which reduces cognitive load in evidence application through structured tools (12). At the adopter level, we innovatively integrated telemedicine, online platforms, and VR/AR technologies, breaking the time-space constraints of traditional patient education while addressing the shortage of healthcare training resources.For instance, the application of virtual reality technology to alleviate preoperative anxiety in patients aligns with recent international research trends on “immersive technologies improving perioperative experiences” (14). At the practical level, establishing specialized nursing teams and multidisciplinary collaboration frameworks has optimized human resource allocation, addressing the challenge of conflicting traditional concepts with innovative cultural practices. These strategies demonstrate a combination of systematic approaches (covering the entire process) and innovative elements (introducing new technologies), providing fresh perspectives for implementing clinical guidelines.

### 4.3 Potential challenges in practical application

While the intervention strategy in this study demonstrates theoretical soundness, it faces multiple practical challenges. First, implementing the multidisciplinary collaboration framework requires institutional backing from hospital leadership. Without proper policy support, ambiguous responsibilities could lead to inefficient execution. Second, adopting telemedicine and VR/AR technologies demands substantial hardware investment and technical training—requirements that may prove challenging for resource-constrained primary care facilities. Third, patient self-management manuals ‘compliance rates might be affected by cultural literacy gaps or varying health literacy levels, necessitating tailored educational support. These issues highlight that intervention effectiveness depends not only on program design but also on dynamic adjustments aligned with hospitals’ actual resources and policy environments.

### 4.4 Limitations and future prospects of the study

While this study has achieved certain progress in developing and implementing guideline application programs, several limitations remain. First, the limited sample size, primarily focusing on four hospitals in Changsha City, may not fully represent national conditions. Second, although multiple intervention strategies were employed, their effectiveness in practice might be constrained by hospital-specific environments and policies. Additionally, long-term outcomes require further tracking and evaluation. Future research could expand the sample size to include more regions and types of medical institutions to validate the study's generalizability. Furthermore, innovative interventions such as AI and big data-driven optimization of nursing processes for improved quality and efficiency should be explored. In-depth investigation into leadership mechanisms in advancing evidence-based practices could provide theoretical support for developing more effective strategies. Beyond sample size and geographical limitations, the study's long-term impact on intervention strategies requires attention. For instance, the sustained effects of training programs on nursing staff behavior and long-term tracking of telemedicine's impact on patient rehabilitation quality remain unverified. Future research could extend to broader regions and include institutions at different levels to validate program universality. Additionally, applications of AI technologies like intelligent decision support systems in guideline updates and personalized recommendations could enhance nursing efficiency. Moreover, leadership and organizational culture's role in promoting evidence-based practices merits deeper exploration, particularly how incentive mechanisms can strengthen healthcare workers’ motivation for change.

### 4.5 Implications for clinical practice and policy making

The guideline implementation framework developed in this study provides a standardized model for ERAS care practices in hepatobiliary surgery, offering replicable optimization pathways for resource-constrained regions. Policymakers can adopt the multidisciplinary collaboration model and technology integration strategies demonstrated in this research to advance regional ERAS care network development. Meanwhile, hospital administrators should prioritize cultivating an innovation culture by implementing incentive mechanisms that encourage nursing staff to actively apply the guidelines. This dual approach ultimately enhances both patient rehabilitation outcomes and the efficiency of medical resource utilization.

Through these studies and practices, we expect to further promote the application of clinical practice guidelines in the field of nursing, improve the quality of care, promote the rapid recovery of patients, and ultimately achieve the optimal allocation of medical resources and the maximization of patient health.

## Data Availability

The original contributions are included in the article/Supplementary Material, and further inquiries can be directed to the corresponding author.
